# Mechanical forces orchestrate the epigenetic landscape of oral mesenchymal stem/progenitor cell fate in dental and periodontal tissues

**DOI:** 10.3389/fcell.2026.1743397

**Published:** 2026-02-27

**Authors:** Yikun Zhou, Gengming Zhang, Hong He

**Affiliations:** 1 State Key Laboratory of Oral & Maxillofacial Reconstruction and Regeneration, Key Laboratory of Oral Biomedicine Ministry of Education, Hubei Key Laboratory of Stomatology, School & Hospital of Stomatology, Wuhan University, Wuhan, China; 2 Department of Orthodontics, School and Hospital of Stomatology, Wuhan University, Wuhan, China

**Keywords:** chromatin remodeling, cytoskeletal mechanics, epigenetic landscape, mechanical forces, oral mesenchymal stem/progenitor cell

## Abstract

The oral cavity serves as the primary source of oral mesenchymal stem/progenitor cell populations residing in the dental pulp, periodontal ligament, deciduous tooth pulp, and gingival connective tissue. Oral and periodontal tissues exist in a constantly loaded biomechanical environment, where forces from mastication, vascular pulsation, and orthodontic manipulation continuously act on resident mesenchymal stem cells, including dental pulp stem cells (DPSCs), periodontal ligament stem cells (PDLSCs), stem cells from human exfoliated deciduous teeth (SHEDs), and gingival mesenchymal stem cells (GMSCs). In this review, we use the term “oral stem cells” to specifically denote oral mesenchymal stem/progenitor populations residing in dental pulp, periodontal ligament (PDL), deciduous tooth pulp, and gingival connective tissue (DPSCs, PDLSCs, SHEDs, and GMSCs), which are most relevant to orthodontic remodeling and dento-periodontal regeneration. For clarity, this review highlights the defining characteristics, representative markers, differentiation potential, and immunomodulatory properties of these oral stem cells within the manuscript, establishing a foundation for understanding how mechanical forces shape their fate. These forces are not merely physical stimuli; they actively reshape stem cell fate by engaging a multilayered mechano - epigenetic regulatory network that integrates cytoskeletal mechanotransduction, nuclear mechanics, and chromatin remodeling. Mechanical inputs such as compression, tension, shear stress, and extracellular matrix stiffness modulate DNA methylation, histone acetylation and methylation, 3D genome architecture, and non-coding RNA programs. These epigenetic and epitranscriptomic adaptations stabilize lineage commitment, influence inflammatory and regenerative outputs, and may establish “mechanical memory” that persists after load removal. Metabolic rewiring, including YAP/TAZ- and MAPK-driven control of mitochondrial activity and metabolite pools, provides an additional axis linking mechanics to chromatin state. Building on these mechanisms, emerging therapeutic strategies aim to couple defined mechanical cues with epigenetic modulators and mechano-tunable biomaterials to enhance pulp regeneration, periodontal repair, and orthodontic bone remodeling with higher precision. The review further highlights single-cell multi-omics and live-cell imaging approaches as essential tools to resolve force-dependent chromatin dynamics *in vivo*, and proposes that integrating biomechanics, epigenetics, and metabolic control will enable next-generation regenerative dentistry and personalized orthodontic intervention.

## 1 Introduction

### 1.1 Oral mesenchymal stem/progenitor cell in dental tissue homeostasis and regeneration

In this review, “oral stem cells” refers specifically to oral mesenchymal stem/progenitor cells (OMSPCs) residing in dental pulp, PDL, deciduous tooth pulp, and gingival connective tissue. The best-characterized OMSPC populations discussed here include DPSCs, PDLSCs, SHEDs, and GMSCs, due to their accessibility, multipotency, and translational relevance in orthodontic remodeling and dento-periodontal regeneration (Sui et al., 2025), which is shown in Table 1.

**TABLE 1:** Characteristics of OMSPC types in dental and periodontal tissues.

Stem cell type	Tissue source	Representative markers (+/−)	Key differentiation potential (reported)	Representative immunomodulatory properties (reported)	Key references
DPSCs	Permanent tooth dental pulp	+ CD29/CD44/CD90/STRO-1; − CD34/CD45	Odontogenic/osteogenic; adipogenic; chondrogenic; neural-like	TGF-β/PGE_2_/IDO secretion; HLA-G/HGF; PBMC suppression	(Gronthos et al., 2002; Gronthos et al., 2000; Özdemir et al., 2016; Pierdomenico et al., 2005; Makino et al., 2013; Liu et al., 2025a; Cao et al., 2020)
PDLSCs	Periodontal ligament	+ CD29/CD44/CD90/CD105/STRO-1; − CD34/CD45	Cementoblastic/fibroblastic/osteogenic adipogenic	IL-6/8-related immune modulation; Treg induction; altered in inflammation	(Yamashita et al., 2024; Oka et al., 2012; Wang et al., 2024; Seki et al., 2023)
SHED	Deciduous tooth pulp	+ CD29/CD44/CD90 − CD34/CD45	Odontogenic/osteogenic; adipogenic; chondrogenic; neural-like	Exosomes suppress Th1 (miR-29a-3p/T-bet); antioxidant effects	(Ng et al., 2016; Laino et al., 2006; Ding et al., 2025a)
GMSCs	Gingival connective tissue	+ CD29/CD44/CD73/CD90/CD105/STRO-1 − CD34/CD45	Osteogenic; adipogenic; chondrogenic; neural-like; epithelial-like (induction)	T- cell suppression; U- Treg induction (CD39/CD73–adenosine); M2 polarization	(Sonoyama et al., 2008; Katahira et al., 2025; Du et al., 2025; Tolouei et al., 2023; Zhang et al., 2021; Li et al., 2024a)

We acknowledge that the term “oral stem cells” can also encompass additional progenitor compartments, including self-renewing basal oral epithelial progenitors and salivary gland stem/progenitor cells, which are essential for mucosal and glandular homeostasis and regeneration (Faraji et al., 2025). However, because the present review aims to synthesize mechanical microenvironment–driven epigenetic regulation in connective-tissue–associated mesenchymal progenitors that are directly implicated in orthodontic remodeling and dento-periodontal repair, we do not comprehensively cover epithelial or salivary gland lineages here. We highlight these populations as important extensions for future mechano-epigenetic studies in the oral cavity.

DPSCs, isolated from the dental pulp of permanent teeth, exhibit strong odontogenic capacity. *In vitro* and transplantation/ectopic models have shown that DPSCs can differentiate into odontoblast-like cells and generate mineralized matrices resembling reparative dentin. Importantly, recent *in vivo* lineage-tracing work demonstrates that Mx1-labeled pulp progenitors are a major source of odontoblast-like cells and contribute substantially to reparative dentinogenesis after molar injury, providing direct evidence for endogenous pulp progenitor function during repair (Yang D. et al., 2025; Gronthos et al., 2002; Gronthos et al., 2000; Cao et al., 2024). DPSCs also exert immunosuppressive activity by secreting soluble mediators such as TGF-β, PGE_2_, and IDO, and by maintaining the expression of immunomodulatory genes including HLA-G and HGF (Özdemir et al., 2016; Pierdomenico et al., 2005; Makino et al., 2013; Liu Y. et al., 2025). Functionally, they inhibit the proliferation of allogeneic peripheral blood mononuclear cells (PBMCs) and reduce TNF-α expression in lipopolysaccharide (LPS)-stimulated THP-1 cells, indicating both anti-inflammatory and tolerogenic properties. (Cao et al., 2020).

PDLSCs, derived from the PDL, are central to periodontal tissue maintenance. They can differentiate into cementoblasts, fibroblasts, and osteoblasts, thereby contributing to the regeneration of cementum, PDL, and alveolar bone (Yamashita et al., 2024; Oka et al., 2012; Cianci et al., 2016; Papagerakis et al., 2014). Consistent with these roles, recent *in vivo* lineage-tracing and mechanically relevant models (e.g., orthodontic tooth movement) support that defined PDL progenitor pools can differentiate into osteoblast/fibroblast lineages in a spatially regulated manner, strengthening the link between PDLSC identity and periodontal remodeling *in situ* (Wang et al., 2024; Seki et al., 2023). Under inflammatory conditions, PDLSCs modulate polymorphonuclear neutrophil (PMN) survival and bactericidal activity through IL-6 and IL-8 secretion, and they promote regulatory T cell (Treg) induction to limit excessive inflammation (Cianci et al., 2016; Ng et al., 2016; Wang Q. et al., 2017). Notably, PDLSCs isolated from inflamed tissues display impaired immunosuppressive capacity, underscoring how the local microenvironment shapes their functional phenotype (Shang et al., 2021).

SHEDs are obtained from the residual pulp of naturally exfoliated deciduous teeth, typically from 6 to 10-year-old children, and can be collected non-invasively without additional donor-site morbidity, making them an attractive autologous source (Miura et al., 2003). SHEDs display high proliferative and clonogenic potential (Miura et al., 2003; Laino et al., 2006; Ding J. et al., 2025). They express mesenchymal markers (CD29, CD44, CD90) and lack hematopoietic markers (CD34, CD45) (Shang et al., 2021). SHEDs can also differentiate into odontoblast-like cells (Sonoyama et al., 2008). Beyond their differentiation potential, SHEDs exhibit immunomodulatory and antioxidant functions: SHEDs-derived exosomes suppress Th1 responses via the miR-29a-3p/T-bet axis, and SHEDs-conditioned medium reduces reactive oxygen species (ROS) (Katahira et al., 2025; Du et al., 2025). In addition, SHEDs support vascular stabilization and promote pulp regeneration (Ding J. et al., 2025).

GMSCs are isolated from the lamina propria of gingival tissues-including free gingiva, attached gingiva, and supracrestal gingiva-and are typically obtained from discarded tissue during routine dental procedures without additional donor morbidity, offering a readily accessible and minimally invasive stem cell source (Grawish, 2018; Srithanyarat et al., 2023; Zhang et al., 2025). GMSCs display a canonical mesenchymal stem cell (MSC) immunophenotype: they are positive for CD29, CD44, CD73, CD90, CD105, and STRO-1, and negative for the hematopoietic markers CD34 and CD45, consistent with an MSC identity (Shetty et al., 2025; Tolouei et al., 2023). These cells exhibit broad differentiation plasticity (Zhang et al., 2021). Under epithelial induction conditions, GMSCs acquire epithelial-like characteristics and express keratin markers (KRT12, KRT19) as well as E-cadherin, in part through inhibition of Wnt/β-catenin signaling (Li et al., 2024a). Owing to their cranial neural crest origin, they can also differentiate into osteogenic, adipogenic, chondrogenic, and neural-like lineages (Tolouei et al., 2023; Huang et al., 2023). Functionally, GMSCs possess strong immunoregulatory activity: they suppress CD4^+^ T cell proliferation and Th17 activation; induce CD4^+^CD25^+^Foxp3^+^regulatory T cells (Tregs) through the CD39/CD73–adenosine axis; and promote M2 macrophage polarization by upregulating CD206 and IL-10, thereby attenuating inflammatory responses (Kim et al., 2021; Wu et al., 2020). Engineered CCR6^+^nanovesicles derived from GMSCs can home to CCL20-rich inflamed tissues and further enhance anti-inflammatory efficacy in autoimmune skin disease models (Huang et al., 2023).

In tissue repair, GMSCs promote regeneration. When encapsulated in a Nap-GDFDFpDY (pY-Gel) supramolecular hydrogel, GMSCs accelerate healing of radiation-induced cutaneous injury by activating the EGFR/STAT3 pathway, which supports cell proliferation, migration, and DNA damage repair (Nie et al., 2022). In periodontal defect models, GMSC-seeded scaffolds generate new cementum, PDL, and alveolar bone, demonstrating their therapeutic relevance for periodontal regeneration (Shetty et al., 2025).

While a substantial body of work has characterized dental mesenchymal stem/progenitor populations using *in vitro* culture systems and transplantation/heterotopic assays, recent *in situ* and *in vivo* approaches are beginning to define their endogenous identities and functions. In the dental pulp, Mx1-based lineage tracing provides direct evidence that a defined pulp progenitor pool contributes markedly to odontoblast-like cell replenishment and reparative dentinogenesis following tooth injury (Yang D. et al., 2025). In parallel, single-cell transcriptomic studies have refined the cellular heterogeneity of dental pulp and periodontal compartments, enabling the identification of progenitor-like subsets and their predicted differentiation trajectories under homeostatic and regenerative contexts. Notably, recent work combining single-cell profiling with functional assays further indicates that PDGFRA^+^ progenitors can orchestrate angiogenesis-coupled periodontal tissue regeneration, highlighting an emerging “*in vivo*” framework for linking progenitor states to repair outcomes (Liu J. et al., 2025). Collectively, these data strengthen the physiological grounding of the dental stem/progenitor populations discussed here and motivate the subsequent sections on how the mechanical microenvironment shapes their fate decisions.

Because specifying tissue origin (e.g., dental pulp-derived or PDL-derived) already denotes the source, this review focuses on mesenchymal stem/progenitor populations from the dental pulp, PDL, deciduous tooth pulp, and gingival connective tissue (DPSCs, PDLSCs, SHEDs, and GMSCs) (Seki et al., 2023; Pittenger et al., 1999; Zuk et al., 2001; Shi et al., 2001; Alge et al., 2010). The epigenetic landscape comprises dynamic DNA and chromatin features—including DNA methylation, histone acetylation/methylation, and chromatin compaction—that shape cell fate decisions. In oral mesenchymal stem/progenitor cells, mechanical cues can reshape this landscape by shifting the balance between euchromatin and heterochromatin, altering chromatin accessibility and regulating lineage-specific gene expression. Here we discuss how oral-relevant mechanical cues can shift these features in dental/periodontal mesenchymal progenitors.

Mechanical stimuli also converge on cellular metabolism. The ‘metabolo-epigenetic axis’ highlights how biomechanical forces modulate mitochondrial activity and metabolic pathways, altering the availability of metabolites such as acetyl-CoA, S-adenosylmethionine and α-ketoglutarate (αKG). These metabolites serve as substrates or cofactors for DNA-and histone-modifying enzymes; by adjusting their levels, mechanical cues indirectly regulate epigenetic states and downstream fate specification. When similar metabolo-epigenetic mechanisms are referenced from non-oral systems, they are presented only as hypothesis-generating context until directly validated in oral stem/progenitor cells.

Accordingly, throughout this review, we prioritize mechanistic studies supported by oral/dental tissues (e.g., PDLSCs, DPSCs, SHEDs, and GMSCs) and explicitly indicate the experimental context (*in situ*/*in vivo* vs. *in vitro*). When concepts are introduced from non-oral MSCs or other cell systems, they are presented only as hypothesis-generating frameworks and are clearly labeled as extrapolations pending validation in oral stem cells.

Nevertheless, despite substantial progress in defining the regenerative and immunomodulatory functions of these oral stem cell populations, the epigenetic programs that govern their fate remain incompletely understood. In particular, how extrinsic cues-most notably mechanical forces-reshape transcriptional states and lineage decisions is still largely unresolved. This gap is critical, because the oral cavity is a mechanically dynamic environment in which physical forces act as constant regulators of tissue behavior, pointing to an urgent need to define how mechanical stimuli orchestrate oral stem cell epigenetics.

### 1.2 The mechanical microenvironment of the oral cavity

#### 1.2.1 Overview of mechanical cues in the oral cavity: definitions, experimental paradigms, and readouts

**FIGURE 1:**
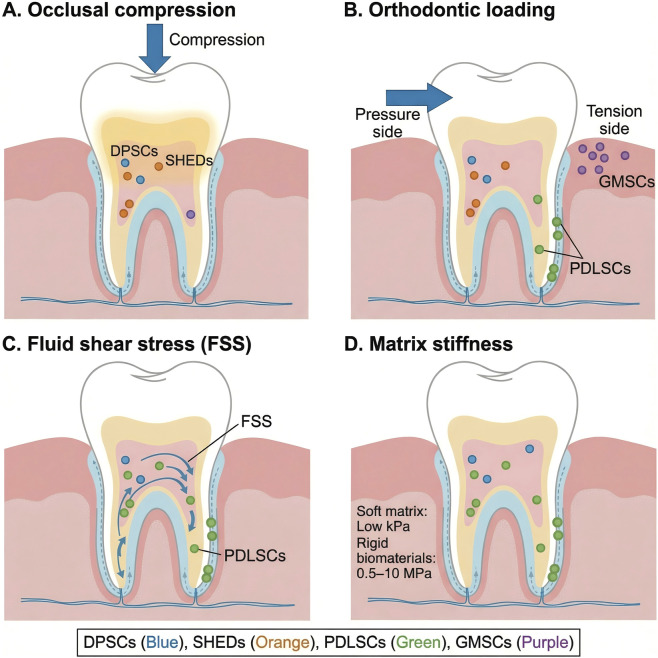
The Oral Mechanical Microenvironment and Resident Stem Cell Populations **(A)** Occlusal compression acting on the pulp (DPSCs/SHEDs) (Hung et al., 2011; Ozcan et al., 2016; Lee et al., 2008; Li et al., 2021); **(B)** Orthodontic loading generating tension and compression in the PDL (PDLSCs/GMSCs) (Bryniarska-Kubiak et al., 2024; Ding R. et al., 2025); **(C)** Fluid shear stress in the PDL/pulp (Shi et al., 2022; Zheng et al., 2016); **(D)** Matrix stiffness differences between soft pulp-like tissues and rigid biomaterials. DPSCs, dental pulp stem cells; SHEDs, stem cells from human exfoliated deciduous teeth; PDL, periodontal ligament; PDLSCs, periodontal ligament stem cells; GMSCs, gingival mesenchymal stem cells.

Mechanical cues in the oral cavity can be categorized into (i) stress-based inputs (compression/pressure, tensile stress), (ii) strain-based inputs (static or cyclic tensile strain), (iii) material property cues (matrix stiffness/viscoelasticity and topography), and (iv) flow-derived cues (fluid shear stress, FSS), interstitial flow) For each cue, it is critical to distinguish the biophysical quantity being controlled—stress (Pa), strain (%), Young’s modulus (Pa), or shear stress (Pa)—from the biological readouts, which commonly include proliferation, migration, inflammatory cytokine production, osteogenic/odontogenic markers, and lineage trajectories assessed by transcriptomic/epigenomic profiling (Yang et al., 2018; Han et al., 2008; Engler et al., 2006; Bertani et al., 2023; Yang et al., 2024a). *In vivo*, occlusal loading and orthodontic tooth movement provide physiologically relevant multi-cue contexts (Wang et al., 2024; Yang et al., 2024b; Rizk et al., 2023)., whereas *in vitro* systems typically isolate one variable using compression rigs, Flexcell-based stretch, tunable hydrogels, or microfluidic perfusion platforms (Yang et al., 2018; Feng et al., 2024; Lu et al., 2015; Mishra et al., 2023) (Table 2). The oral mechanical microenvironment and resident stem cell populations are shown in Figure 1.

**TABLE 2:** Mechanical cue → pathways → outcomes in OMSPCs (with evidence level) Mechanical forces reshape the epigenetic state.

Cell type	Mechanical cue (report unit)	Key pathways/Molecules	Phenotypic output	Evidence level (oral OMSPC vs. extrapolated)	Key references
DPSCs	Strain (%/Hz); stiffness (kPa/MPa); FSS (dyn/cm^2^)	Nrf2/HO-1; stiffness-linked odontogenic markers	Inflammation/antioxidant response; odontogenic/osteogenic bias	Mostly in vitro oral	(Mishra et al., 2023; Hung et al., 2011; Ozcan et al., 2016; Yu et al., 2009; Kraft et al., 2010; Samiei et al., 2023)
PDLSCs	Compression/strain; cyclic tension; FSS	NAT10–ac4C(BMP2 mRNA); p38–AMOT–YAP; MAPK/Wnt/TGF-β	Osteogenesis/proliferation; remodeling programs	Strong in vitro oral; in vivo context exists (OTM)	(Yang et al., 2024b; Bryniarska-Kubiak et al., 2024; Shi et al., 2022; Sun et al., 2022; Huang et al., 2018; Chen et al., 2025; Suwittayara et al., 2025)
SHED	Physiologic resorption context; engineered topography/stiffness	α7nAChR/SLURP-1 axis (reported); mechanosensors not yet directly validated	Root-resorption-related programs; differentiation shifts	Oral but mechanosensor evidence limited	(Ng et al., 2016; Wang et al., 2017a; Shang et al., 2021; Eichholz et al., 2020; Zheng et al., 2016)
GMSCs	Force-associated models; tension– immune crosstalk	M2-exo–MeCP2–TCF20–HDAC1; Wnt/β-catenin de-repression	Osteogenesis + immunomodulation	Oral-related mechanistic axis	(Tolouei et al., 2023; Zhang et al., 2021; Li et al., 2024a; Huang et al., 2018; Hodge et al., 2011)

#### 1.2.2 Mechanical cues and loading conditions

Mechanical forces act as core regulators of oral stem cell fate: compression, extracellular matrix (ECM) stiffness, and scaffold-mediated tension drive distinct phenotypic shifts and functional adaptations that shape regenerative outcomes. These cues frequently interface with epigenetic programs to stabilize lineage decisions.

Oral and periodontal mesenchymal stem/progenitor cells integrate compression/pressure, tensile strain, fluid shear stress, matrix stiffness/topography, and hydrostatic pressure/curvature to shape fate decisions through a conserved mechanotransduction network centered on YAP/TAZ, MAPK (p38/ERK1/2), Wnt/β-catenin, BMP/TGF-β–Smad, RhoA/ROCK, mechanosensitive ion channels (Piezo1/TRPV4), inflammatory signaling (NF-κB), and cytoprotective pathways such as Nrf2/HO-1, with epitranscriptomic/epigenetic coupling reinforcing downstream programs. In DPSCs, compression and stiffness cues promote proliferation, ECM remodeling, and anti-apoptotic/antioxidant responses, while compliant matrices favor stemness maintenance and stiff matrices enhance DSPP expression and mineralization. PDLSCs are highly responsive to cyclic stretch and shear, where stimulus magnitude switches between stemness preservation (low FSS) and osteogenesis (high FSS); compression can further drive osteogenic commitment in PDLSCs; the underlying epitranscriptomic mechanism is described in a later section, supporting PDL/cementum regeneration and anti-inflammatory remodeling. SHED respond to dynamic compression, micro/nanotopography, and tension with odontogenic/osteogenic, angiogenic, and neurogenic potentials, with compression biasing toward osteoclastogenic/root-resorption programs whereas tension enhances proliferation and osteogenic markers (OPN, Col1). GMSCs under tensile strain and stiffness shifts exhibit myofibroblast/wound-healing and anti-fibrotic phenotypes, and may promote osteogenesis through an M2-exosome–MeCP2–TCF20 pathway that relieves histone deacetylases 1(HDAC1) repression of Wnt/β-catenin. DFSCs interpret hydrostatic pressure and curvature through integrin–cytoskeleton coupling and Rho/ROCK/MLC–YAP/TAZ–Wnt signaling to promote periodontal regeneration, angiogenesis, neural crest specification, and tissue remodeling, supported by both *in vitro* and *in vivo* models.

Human dental pulp is a non-mineralized, extremely soft tissue (Hung et al., 2011). Uniaxial compression (10% strain, 0.05 mm min^-1^) yields a compressive modulus of ∼5.5 kPa (5.5 ± 2.8 kPa), while stress-relaxation tests reveal marked viscoelasticity: after 20% compression, stress decays to a negligible residual level (0.26 ± 1.48 kPa) at 1,000 s, underscoring its softness (Ozcan et al., 2016). Consequently, even regular mastication imposes physiologic loads on the pulp, Cyclic strain that mimics masticatory stress induces human dental pulp cells (HDPCs; DPSC-like) to produce inflammatory cytokines (IL-6, IL-1β, TNF-α) and antioxidant enzymes (HO-1, SOD) (Lee et al., 2008; Lee et al., 2010). Limited physiological loading stimulates reparative odontoblast activity and tertiary dentinogenesis. In intact pulp, a ∼1–10 kPa range (if known) hydrogel-like 3D ECM niche helps maintain DPSC stemness, whereas materials with MPa-range modulus (e.g., ≥0.5 MPa) scaffolds or high-pressure bias cells toward osteo/odontogenic differentiation (Lee et al., 2008; Lee et al., 2010; Bryniarska-Kubiak et al., 2024).

Orthodontic appliances impose sustained tensile and compressive forces on the tooth–PDL complex. Within the PDL, static compression (pressure side) promotes bone resorption, whereas tension (opposite side) drives bone formation (Feng et al., 2024; Li et al., 2021).

#### 1.2.3 Mechanosensing apparatus

Mechanical inputs are first detected by a mechanosensing apparatus that includes integrin–focal adhesion complexes, the actin cytoskeleton, mechanosensitive ion channels (e.g., Piezo-family channels and TRPV4), and mechano-coupling to the nucleus.

DPSCs are regulated by multiple mechanical cues, including FSS, matrix stiffness, and compressive loading, which together shape lineage specification and functional output (Yan et al., 2025). *Stiffness cues should be reported as absolute modulus values rather than relative labels.* In this review, we therefore separate kPa-range compliant matrices (e.g., soft hydrogels and polymeric scaffolds, typically ∼1–50 kPa) from MPa-range rigid biomaterials (e.g., mineralized or cement-like constructs, typically ∼0.5–10 MPa). This avoids misleading cross-study comparisons in which “high stiffness” within a hydrogel system (e.g., 18–40 kPa) is still orders of magnitude lower than MPa-scale materials (e.g., 5–7 MPa = 5,000–7,000 kPa). Where possible, we provide direct unit conversions (1 MPa = 1,000 kPa) and specify whether reported values refer to Young’s modulus or compressive modulus, as these metrics may differ across platforms (Bryniarska-Kubiak et al., 2024; Qu et al., 2015; Vahabzadeh et al., 2020).

PDLSCs inhabit a mechanically dynamic niche shaped by compression, tension, FSS, and matrix stiffness. These forces are sensed through focal adhesions, the actin cytoskeleton, and mechanosensitive ion channels, and transmitted via the cytoskeletal network to the nucleus. FSS in the range of ∼one to six dyn/cm^2^ rapidly reorganizes F-actin into a perinuclear cap and flattens the nucleus, illustrating how cytoskeletal and nuclear mechanics cooperate during force sensing (Shi et al., 2022). Cyclic tensile strain has been shown to promote proliferation and osteogenic output in DPSCs, Although direct evidence in SHEDs is limited, their shared mesenchymal origin and regenerative capacity suggest they may exhibit a similar mechanoresponsive trend. In osteocyte research, fluid shear stress–induced NO and PGE signaling has been shown to promote stem cell chemotaxis and osteogenic differentiation. Considering the behavior of DPSCs, mechanical stimulation may trigger similar signaling pathways, enhancing their functional consistency with bone marrow–derived mesenchymal stem cells (Eichholz et al., 2020; Yu et al., 2009).

#### 1.2.4 Downstream signaling pathways

Upon sensing, mechanical inputs are converted into biochemical signaling through canonical mechanotransduction cascades and lineage-associated pathways. In DPSCs, mechanical strain activates the Nrf2/HO-1 stress-response pathway, coupling antioxidant signaling to odontogenic differentiation (Lee et al., 2008; Lee et al., 2010). FSS designed to mimic masticatory flow further modulates DPSCs in a maturation-dependent manner: more committed DPSCs display enhanced osteogenic potential and can generate organized lamellar bone *in vivo* under hydrodynamic stimulation (Kraft et al., 2010). DPSCs also respond to imposed compressive and shear forces through engineered biomaterials. A compressive load of ∼9.7 MPa applied in the PNIPAAm–GO–CS hydrogel system upregulated osteogenic markers and accelerated mineralization (Samiei et al., 2023). Three-dimensional β-tricalcium phosphate scaffolds, tuned to approximate bone stiffness, enhance adhesion, differentiation, and mineral nodule formation, underscoring the instructive role of scaffold mechanics in directing DPSC fate (Pérez-Sánchez et al., 2025). Nonetheless, current *in vitro* systems do not fully recapitulate the complex, cyclic, multiaxial loading environment experienced *in vivo* within dentinal tubules and the vascularized pulp (Sun et al., 2022).

In PDLSCs, FSS activates p38 MAPK and initiates an Akt–cofilin–YAP cascade that promotes proliferation (Shi et al., 2022). Orthodontic stretch engages MAPK, Wnt/β-catenin, and TGF-β/Smad signaling, with mediators such as IL-11 and miR-21 coordinating osteogenic differentiation and inflammatory tone (Huang et al., 2018). Orthodontic compression further drives osteogenic commitment in PDLSCs through post-transcriptional regulation. Specifically, the acetyltransferase NAT10 increases N^4^-acetylcytidine (ac4C) on BMP2 mRNA, stabilizing BMP2 and elevating osteogenic markers including RUNX2 and Osterix (Feng et al., 2024). In parallel, M2 macrophage–derived exosomes under tension activate the MeCP2–TCF20 complex in PDLSCs, which relieves HDAC1-mediated repression of Wnt/β-catenin signaling and promotes osteogenesis (Chen et al., 2025). The magnitude of FSS also matters: low FSS (0.5 dyn/cm^2^) supports survival and stemness programs, whereas higher FSS (6 dyn/cm^2^) induces osteogenic markers such as ALP and OPN (Suwittayara et al., 2025; Zheng et al., 2016).

In SHEDs, chewing-like cyclic compression *in vitro* upregulates SLURP-1 and α7 nicotinic acetylcholine receptors, activates NF-κB signaling, and promotes osteoclastogenic differentiation associated with physiological resorption (Wang L. et al., 2017).

**FIGURE 2:**
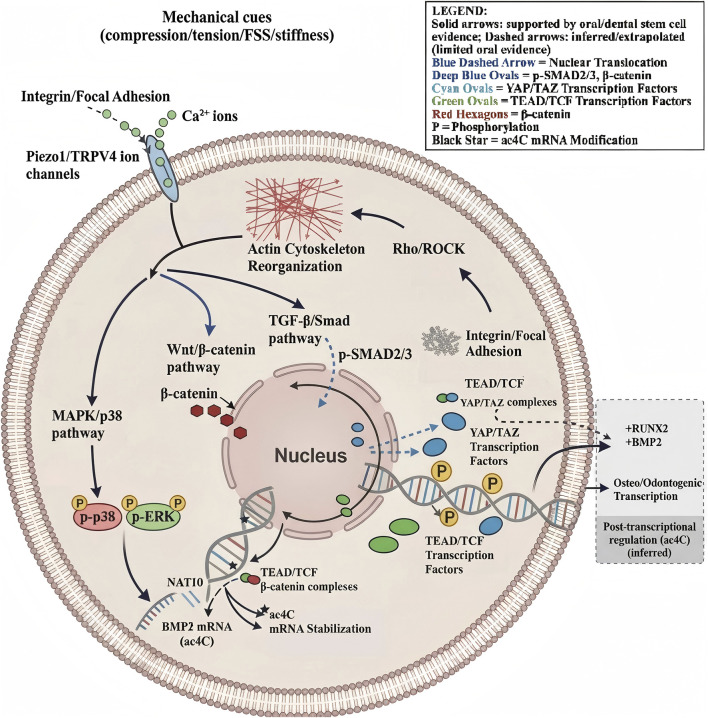
Integrated core mechanotransduction pathways: provide a stepwise description from mechanical cues → membrane/ion-channel/focal adhesion sensing → cytoskeletal and kinase pathways (Rho/ROCK, MAPK, TGF-β/Smad, Wnt/β-catenin) → nuclear translocation of YAP/TAZ (Shi et al., 2022), β-catenin, p-SMAD2/3 → transcriptional and RNA-modification outputs (e.g., NAT10-mediated ac4C on BMP2 mRNA) (Feng et al., 2024). FSS, low fluid shear stress; ROCK, Rho-Associated Protein Kinase; MAPK, Mitogen-Activated Protein Kinase; TGF-β, transforming growth factor-β; Smad, Smad Interaction Domain; Wnt, wingless/integrated; YAP, yes-associated protein; TAZ, transcriptional co-activator with PDZ-binding motif; RNA, ribonucleic acid; NAT10, N-acetyltransferase 10; ac^4^C, N^4^-acetyl cytidine; BMP2, bone morphogenetic protein two messenger; ERK, extracellular signal-regulated Kinase; TEAD/TCF, transcriptional enhanced associate domain protein/T-cell factor; Piezo1/TRPV4, piezo-type mechanosensitive ion channel component 1/transient receptor potential cation channel subfamily V member 4; RUNX2, runt-related transcription factor 2.

In GMSCs, orthodontic tension promotes M2 macrophage polarization, and M2-derived exosomes activate a MeCP2–TCF20 complex in GMSCs, relieving HDAC1-mediated repression of Wnt/β-catenin and driving osteogenesis (Chen et al., 2025). Mechanical cues in GMSCs are therefore likely decoded through pathways analogous to those in PDLSCs, including YAP-mediated mechanotransduction, inflammatory modulation, and potential osteogenic specification—supported by their shared responsiveness to substrate stiffness, conserved YAP-dependent mechanosignaling, and the established link between matrix rigidity, inflammatory regulation, and lineage specification in oral tissue-derived mesenchymal stem cells (Tiskratok et al., 2023). These pathways are inferred from PDLSCs and other mesenchymal systems; direct validation in GMSCs under mechanical loading is still needed. The operating mechanism of intracellular mechanical signal transduction pathway is briefly summarized in Figure 2.

#### 1.2.5 Epigenetic mechanisms encoding mechanical signals

Downstream signaling is ultimately “written” into chromatin through epigenetic mechanisms, including histone modifications, DNA methylation, non-coding RNAs, and nuclear–chromatin architectural regulation. In developmental biology, C. H. Waddington proposed the “epigenetic landscape” as a metaphor to explain how pluripotent cells commit to specific lineages. In this model, cell fate is represented by a ball rolling down a landscape of hills and valleys: the ball’s initial position is multipotent, while the valleys correspond to stable differentiated states and the ridges represent epigenetic barriers that restrict transitions. The topology of this landscape is sculpted by gene regulatory networks and epigenetic marks such as DNA methylation, histone modifications, chromatin compaction, and non-coding RNAs (Henikoff, 2023; Zhou et al., 2025).

In the context of oral mesenchymal stem cells, mechanical cues act as forces that “tilt” the epigenetic landscape. Compression, tension, shear stress, substrate stiffness, and topographical features can alter the activity of DNA methyltransferases (DNMTs) and demethylases, histone acetyltransferases (HATs), HDACs, and methyltransferases (e.g., Polycomb repressive complex 2, PRC2), as well as remodel 3D genome architecture and nuclear lamina interactions (Yang S. et al., 2025; Li et al., 2024b). These mechanotransductive pathways converge on chromatin, changing the depth and slope of the valleys and thereby biasing stem cells toward odontogenic, osteogenic, or fibrogenic fates. For example, stiff matrices and cyclic tensile strain increase global DNA methylation and histone acetylation in PDLSCs, whereas compressive loading can activate EZH2-mediated H3K27 trimethylation to suppress osteogenic genes (Hu and Fan, 2025).

An important feature of this model is “mechanical memory”: mechanically induced epigenetic states may persist after removal of the stimulus. Sustained expression of mechanosensitive non-coding RNAs and stable changes in chromatin accessibility can maintain the new landscape configuration, predisposing cells to respond differently to subsequent cues (Kloc and Wosik, 2025). This concept has practical implications for tissue engineering, where combining biomechanical design (e.g., scaffold stiffness, dynamic loading regimens) with epigenetic modulators can deliberately tilt the landscape toward regenerative outcomes (Dudaryeva et al., 2023). By understanding and manipulating the mechanobiology of the epigenetic landscape, dental research may develop precision approaches to regenerate pulp and periodontal tissues.

To orient readers, we note that mechanical cues in dental and oral tissues can be stabilized through epigenetic regulation, thereby biasing transcriptional programs and cell-fate trajectories beyond immediate signaling events. In the following sections (Chapters IV–VI), we systematically discuss how specific mechanical inputs are sensed and transduced to distinct epigenetic layers—including DNA methylation, histone modifications, non-coding RNA regulation, nuclear mechanotransduction, and higher-order chromatin organization—and how these mechanisms ultimately shape regeneration- and remodeling-relevant outcomes. An integrative network-level synthesis is provided in Chapter VII.

In DPSCs, substrate stiffness modulates nuclear and chromatin state through histone-regulatory enzymes: HAT1 is upregulated while HDAC1 remains stable, a balance that favors maintenance of stemness and limits senescence, suggesting that the mechanical environment imposes an epigenetic constraint on fate decisions (Ghaffari and Shrestha, 2025). Notably, such mechanically induced chromatin configurations can persist after removal of the original stimulus, indicating a form of “mechanical memory” that biases future responses (Ghaffari and Shrestha, 2025; Cosgrove et al., 2025).

At the level of cis-regulatory DNA, stiffness-responsive enhancers (“mechano-enhancers”) act as control nodes. These elements recruit chromatin-modifying complexes and fine-tune expression of genes governing apoptosis, proliferation, and differentiation; targeted epigenetic editing at these enhancers can rewire how cells interpret matrix stiffness, supporting a causal path from mechanics - chromatin remodeling - transcriptional output (Cosgrove et al., 2025). Beyond stiffness, compressive loading can drive ERK-coupled chromatin remodeling and shifts in histone methylation that rejuvenate aged dermal fibroblasts, enhancing their migratory and pro-regenerative behavior. (Liu H. et al., 2025). This mechano-epigenetic rejuvenation principle, established in dermal models, presents a testable hypothesis for whether similar mechanisms could enhance the regenerative capacity of oral mesenchymal cells exposed to orthodontic forces. By analogy, tension-induced nuclear translocation of YAP/TAZ and their TEAD partnerships offers a mechanosensitive co-regulatory route that could promote dentinogenic programs in DPSCs (Uhler and Shivashankar, 2017).

#### 1.2.6 Fate outcomes-lineage commitment, survival, inflammatory behavior

Together, mechanosensing and downstream signaling converge on chromatin- and RNA-level regulation, resulting in transcriptional reprogramming that governs lineage commitment, survival, and inflammatory behavior.

*In vivo* orthodontic force induces site-specific osteogenic responses in alveolar bone: Osterix, ALP, and osteopontin are upregulated, with Osterix peaking at day 7 and ALP at day 14, particularly on the compression side (Nugraha et al., 2024). Hypoxia-preconditioned GMSCs amplify Osterix expression, suggesting cooperative regulation by mechanical and metabolic cues (Nugraha et al., 2024). Critically, many of these mechanotransductive events converge on chromatin- and RNA-level control, leading directly into the next question: how mechanical forces reshape the epigenetic landscape of oral stem cells.

#### 1.2.7 *In vivo* evidence and limitations

*In vivo* mechanobiology of oral stem cells has been most extensively studied in rodent orthodontic tooth-movement (OTM) systems. In these experiments, nickel–titanium coil springs are surgically placed between the maxillary first molar and incisor to apply controlled forces (Yan T. et al., 2024; Mai et al., 2024). Such loading triggers a rapid aseptic inflammatory cascade in the PDL and surrounding tissues. For example, in mouse OTM models, PDL tissues upregulate pro-inflammatory mediators (TNF-α, IL-1β, IL-6, IFN-γ, PGE_2_) within hours of force application (Wang et al., 2023; Kong et al., 1999). This response recruits CD68^+^ iNOS^+^ M1 macrophages to compression sites; depletion of these macrophages reduces tooth-movement distance, whereas adoptive transfer enhances bone resorption and tooth displacement (Wang et al., 2023). Adaptive immunity also plays a role: T-cell-deficient mice exhibit attenuated OTM, while adoptive transfer of allogeneic T cells restores normal movement and increases RANKL production, promoting osteoclastogenesis (Kong et al., 1999; Horton et al., 1972; Yan et al., 2015). These *in vivo* findings underscore the interplay between mechanical forces, immune cells and bone remodeling during orthodontic loading.

OTM studies reveal distinct cellular behaviors on the compression versus tension sides of the PDL (Wang et al., 2022). On the pressure side, PDLSCs and fibroblasts secrete macrophage colony-stimulating factor (M-CSF) and receptor activator of NF-κB ligand (RANKL) (Hodge et al., 2011; Faulkner et al., 2019), leading to recruitment and activation of osteoclast precursors and subsequent bone resorption. Conversely, mechanical tension enhances vascular perfusion and induces osteoinductive cytokines and growth factors that drive osteoblast differentiation (Nakai et al., 2023; Ubuzima et al., 2024; Abu-Amer, 2013). *In vivo*, osteogenic markers such as type I collagen, osteocalcin and alkaline phosphatase peak on the tension side (Yang L. et al., 2025; Zhong et al., 2024). These spatially segregated responses highlight how mechanical cues orchestrate bone resorption and formation to achieve controlled tooth movement.

Beyond orthodontic models, physiological occlusal forces are crucial for alveolar bone homeostasis. *In vivo* experiments show that alveolar bone is subjected to occlusal forces during mastication and that these forces act through the mechanosensitive ion channel Piezo1 (Yang et al., 2024b; Wang et al., 2025). A recent study in female mice demonstrated that loss of occlusal loading causes alveolar bone loss, whereas activation of Piezo1 rescues this phenotype. The authors identified Piezo1 as an occlusal force sensor in osteoblasts; Piezo1 activation directly promotes osteogenesis and regulates osteoclastic apoptosis via Fas ligand–mediated pathways (Yang et al., 2024b). These findings suggest that Piezo1 mediates occlusal-force-dependent bone formation and may serve as a therapeutic target to prevent alveolar bone loss due to occlusal deficiencies or systemic metabolic disorders.

Despite these advances, *in vivo* mechanistic data are primarily available for PDLSCs. Little is known about how DPSCs, SHEDs or GMSCs respond to mechanical loading in their native environments; most mechanistic insights for these populations derive from *in vitro* or extrapolated studies. Furthermore, few *in vivo* studies directly examine how mechanical forces modify epigenetic states in oral stem cells. There is an urgent need for lineage-tracing models, single-cell multi-omics analyzes and conditional knockouts in rodents to verify whether the signaling pathways described *in vitro* operate *in vivo*. In this review, pathways supported by *in vivo* data are depicted with solid lines, whereas mechanisms inferred from non-oral tissues or culture studies are illustrated with dashed lines and accompanied by cautionary notes. We therefore emphasize that extrapolations from other systems should be interpreted carefully until validated in oral tissues.

## 2 DNA methylation dynamics in mechanoresponsive oral stem cells

This section emphasizes oral/dental evidence and notes non-oral findings only when they provide limited conceptual context. Mechanical forces reshape the epigenetic state of oral mesenchymal stem cells by regulating DNA methylation writers and site-specific CpG methylation. In PDLSCs, orthodontic-like compression elevates DNA methyltransferase (DNMT) activity and induces locus-specific hypermethylation: DNMT1 and DNMT3B are recruited to the MIR31HG promoter, leading to promoter hypermethylation, silencing of this proliferation-regulating lncRNA, and a concomitant increase in IL-6 under load (Han et al., 2021) (Supplementary Table S1).

Force-treated cells show MIR31HG promoter hypermethylation by MassARRAY, and this repression is reversed by DNMT1/3B knockdown or by DNA methyltransferase inhibitor 5-aza-2′-deoxycytidine (5-aza-dC) treatment, establishing a causal DNMT-promoter methylation-ranscript-silencing relationship (Han et al., 2021). Mechanical cues are also likely to influence active demethylation: studies of PDLSC biology note coordinated changes in DNMTs and Ten–eleven translocation (TET) enzymes during osteogenic programming, supporting a dynamic balance between 5-methylcytosine (5 mC) and 5-hydroxymethylcytosine (5hmC) during lineage selection (Davletgildeeva and Kuznetsov, 2024; Cao et al., 2023; Yu et al., 2019). Substrate stiffness can encode “mechanical memory” through global methylation control. In human PDLSCs cultured on stiff GelMA (∼7 kPa), global 5-mC levels increase, total DNMT activity rises, and DNMT3B protein is selectively upregulated. These changes correlate with elevated RUNX2, Col-1, ALP, and alkaline phosphatase activity; pharmacologic DNMT inhibition reduces these osteogenic readouts (Ding R. et al., 2025). Similarly, stiff extracellular matrix drives genome-wide hypermethylation and silencing of pluripotency-associated promoters in mouse stem cells, indicating a conserved stiffness-DNA methylation-fate axis (Zhao et al., 2021).

These DNA methylation programs are functionally targetable (Loyfer et al., 2023; Yang Y. et al., 2025). Under high-glucose conditions, PDLSCs display elevated DNMT expression, global hypermethylation, and impaired osteogenesis; treatment with the DNMT inhibitor 5-aza-dC restores mineralization and osteogenic gene expression by reactivating canonical Wnt/β-catenin signaling (Liu et al., 2016). *In vivo*, diabetic rats exhibit increased 5-mC in the PDL together with alveolar bone loss, linking pathological hypermethylation to defective regeneration (Liu et al., 2016).

Taken together, across PDLSCs and related stem systems, mechanical tension and stiffness coordinate DNMT1/3B-dependent methylation at key loci (e.g., MIR31HG) and elevate global 5-mC, while demethylation pathways (TET/5hmC) appear poised to modulate lineage decisions (Tiskratok et al., 2023; Ghaffari and Shrestha, 2025; Zhang et al., 2022; Li Z. et al., 2020; Jabre et al., 2025). Stiff matrices and pathological stress favor hypermethylation, repress stemness-associated genes, and bias cells toward osteogenic output; conversely, pharmacologic DNA demethylation can rescue osteogenesis via Wnt signaling, highlighting DNA methylation as a tractable effector of mechanotransduction in oral tissues (Cosgrove et al., 2025; Liu H. et al., 2025; Yu et al., 2021).

## 3 Histone post-translational modifications: a mechanosensitive code

We focus on histone-modification changes demonstrated in periodontal/dental stem or stromal cells under defined mechanical cues, and we avoid extending non-oral MSC mechanisms unless oral data are available. Mechanical cues reprogram oral stem-cell fate in part by reshaping histone post-translational modifications (PTMs) (Supplementary Table S2). On stiff matrices, increased nuclear tension suppresses HDAC activity, elevates global histone acetylation, and drives RUNX2 expression and nuclear localization, establishing an epigenetic route toward osteogenic differentiation in human mesenchymal stem cells (Killaars et al., 2020).

Disruption of nucleo–cytoskeletal coupling reverses these effects by restoring HDAC activity, whereas HDAC inhibition rescues histone acetylation and osteogenic programming, indicating a causal role for acetylation in mechanotransduction (Killaars et al., 2020). In PDL cells, cyclic stretch reorganizes actin and tubulin, rapidly increases histone H3 acetylation, and decreases the repressive mark H3K9me3-signatures of a more open chromatin state; similar acetylation changes are observed *in vivo* under altered occlusal loading (Bae et al., 2024). Moreover, in compressed human PDL fibroblasts, increasing H3 acetylation (via HDAC blockade or metabolic cues) elevates the anti-inflammatory cytokine IL-10, whereas histone acetyltransferase (HAT) inhibition prevents this induction, directly linking force, acetylation, and gene activation (Schuldt et al., 2022).

Mechanical stress also retunes histone methylation. In PDLSCs, compressive force downregulates EZH2 and decreases global H3K27me3; preventing EZH2 loss traps this Polycomb-group methyltransferase on chromatin and impairs multipotency, indicating that relief of Polycomb repression is required for a normal mechanoresponse (Li Q. et al., 2020). In parallel, Trithorax-group–associated demethylases promote access to osteogenic genes: studies in dental MSCs show that KDM6-family H3K27 demethylases remove H3K27me3 at promoters such as BMP2, RUNX2, and ALP, coincident with gains in activating marks like H3K4me3 and progression toward osteogenic differentiation (Hu and Fan, 2025; Xu et al., 2013). Thus, mechanical inputs tend to attenuate PRC2–H3K27me3 repression while favoring TrxG-driven activation, aligning chromatin states with the prevailing biomechanical environment (Hu and Fan, 2025; Li Q. et al., 2020).

Emerging acyl modifications reveal a direct metabolism–mechanics–epigenetics axis. During orthodontic tooth movement, tensile force elevates lactate levels and installs histone lactylation in alveolar bone–derived mesenchymal stem cells; inhibition of lactate synthesis suppresses force-induced proliferation and osteogenic gene expression, and these effects are mediated by locus-specific lactylation as mapped by ChIP-seq (Zhai et al., 2022). Similarly, adaptive hydrogels that impose dynamic, hypoxia-like mechanical conditions increase lactate production and H3K18la, with ChIP-qPCR showing enrichment of this mark at chondrogenic genes and enhanced cartilage differentiation in human MSC organoids (Yang B. et al., 2025). Lactylation and acetylation share enzymatic writers and erasers (e.g., p300 and HDACs) and are co-regulated under mechanical stress, suggesting that force-adjusted metabolism can coordinately tune multiple histone acylations (Gong et al., 2024). Related crosstalk with crotonylation and other acyl marks further supports the existence of a broader “acyl code” responsive to biomechanical and metabolic state (Gong et al., 2024).

**FIGURE 3:**
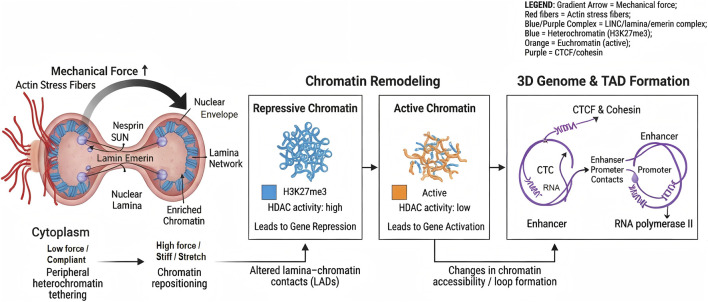
Force-Driven Chromatin Remodeling and Nuclear Mechantronsuction: Force transmission via actin stress fibers and the LINC (Linker of Nucleoskeleton and Cytoskeleton) complex (Nesprin/SUN) → deformation of the nuclear lamina (Lamin A/C, Lamin B1) and changes in lamina-associated domains (LADs) → shifts between repressive (H3K27me3-enriched) and active chromatin (Killaars et al., 2020; Seelbinder et al., 2021) → reorganization of topologically associating domains (TADs) and enhancer–promoter loops mediated by CTCF and cohesin. LINC, linker of nucleoskeleton and cytoskeleton; LADs, lamina-associated domains; TADs, topologically associating domains; H3K27me3, Histone H3 lysine 27 trimethylation; HDAC, histone deacetylase; CTCF, CCCTC-binding factor; TAD, topologically associating domain.

Together, these findings indicate that mechanical forces reconfigure histone acetylation (through HAT/HDAC balance) to open chromatin and activate lineage or immunomodulatory programs (Killaars et al., 2020; Bae et al., 2024; Schuldt et al., 2022), and recalibrate histone methylation by reducing Polycomb-mediated H3K27me3 while enabling Trithorax-driven activation at osteogenic loci (Hu and Fan, 2025; Li Q. et al., 2020; Xu et al., 2013). Concurrently, force-induced metabolic shifts install histone lactylation that licenses regenerative responses (Zhai et al., 2022; Yang B. et al., 2025; Gong et al., 2024). Collectively, this defines a mechanosensitive histone code through which physical cues durably program oral stem-cell fate (Figure 3).

Together, these observations suggest that mechanotransduction not only transmits external forces via YAP/TAZ, MAPK and mechanosensitive ion channels but also reprograms cellular metabolism. Force-induced activation of these pathways modulates mitochondrial function, glycolytic flux and glutaminolysis, thereby shifting the pools of acetyl-CoA, αKG and NAD^+^ that feed chromatin-modifying enzymes. This metabolic rewiring provides a mechanistic link between biomechanical input and epigenetic remodeling, setting the stage for chromatin architecture changes and lineage specification discussed in the following sections.

## 4 Chromatin architecture remodeling under force

### 4.1 Nuclear mechanotransduction and chromatin compaction

This section summarizes mechanistic evidence for chromatin architecture remodeling under force in oral mesenchymal stem/progenitor cells. Findings derived from non-oral systems (e.g., skeletal muscle, cardiomyocytes, fibroblasts) are presented briefly and explicitly labeled as extrapolations requiring direct validation in dental or periodontal stem cells.

Mechanical cues in stem-cell systems relevant to oral tissues influence fate by directly acting on the nucleus and remodeling chromatin architecture (McCreery et al., 2025). Evidence from non-oral models (e.g., skeletal muscle) indicates that the nucleus itself is mechanosensitive: in skeletal muscle, lamin A/C preserves appropriate chromatin accessibility under load, whereas its loss results in aberrant chromatin opening and stress-induced transcriptional dysregulation, illustrating a lamina–chromatin force-sensing axis (Jabre et al., 2025).

In non-oral models such as skeletal muscle and fibroblasts, lamin A/C and emerin mediate force-induced chromatin compaction; these findings offer a conceptual framework for force–heterochromatin coupling, but whether similar mechanisms exist in oral mesenchymal stem cells remains to be experimentally validated. These mechanisms are primarily established in non-oral models and should be interpreted as conceptual frameworks pending validation in oral stem cells. (Jabre et al., 2025; Seelbinder et al., 2021; Heo et al., 2015; Fernandez et al., 2022; Carley et al., 2021).

### 4.2 3D genome reorganization under force: TADs and chromatin looping

High-resolution 3D genome evidence under force is currently scarce in dental/periodontal stem cells; thus, the models discussed below derive mainly from non-oral systems and should be considered hypotheses for future validation in oral tissues. Most mechanistic evidence for force-dependent reorganization of chromosome territories, A/B compartments, chromatin loops, and topologically associating domains (TADs) currently derives from non-oral cell systems (e.g., fibroblasts, epithelial cells, and contractile lineages). Direct, high-resolution 3D genome mapping in oral/dental stem-cell populations under defined mechanical loading remains scarce. Accordingly, unless explicitly stated as oral/dental data, the mechanistic models summarized below should be interpreted as frameworks extrapolated to the oral context and prioritized for future validation.

In non-oral cell systems such as fibroblasts, epithelial cells and contractile lineages, mechanical forces transmitted via the cytoskeleton and nuclear lamina reorient chromosome territories and reposition them radially, generating new chromosomal neighborhoods correlated with transcriptional changes. Tension at the nuclear lamina and phosphorylation of lamin B1 lead to large-scale mixing of euchromatin and heterochromatin and to shifts in A/B compartmentalization. At finer scales, mechanical inputs modulate the cohesin–CTCF loop-extrusion machinery, altering chromatin loop stability and topologically associating domain boundaries and thereby changing enhancer–promoter communication. These reorganized structures coincide with the formation of RNA polymerase II-enriched contact hubs and transcriptional rewiring. Collectively, these observations provide a conceptual framework for force-dependent 3D genome remodeling, but high-resolution mapping under defined mechanical load is currently scarce in dental or periodontal stem cells; therefore, this topic remains an important area for future research (Yang S. et al., 2025; Carley et al., 2021; Wang Y. et al., 2017; Alisafaei et al., 2019; Maharana et al., 2016; Maki et al., 2021; Downing et al., 2013; Heo et al., 2016; Hernandez et al., 2016; Ghosh et al., 2012; Elhanany-Tamir et al., 2012; Wang S. et al., 2015; Mishra et al., 2024). This part of the content is briefly shown in Figure 3. The above findings are derived from non-oral models and should be viewed as hypotheses for future validation in oral tissues. To our knowledge, there is currently no high-resolution 3D genome mapping of dental or periodontal stem cells under mechanical loading; therefore, this topic remains an important area for future research.

### 4.3 ATP-dependent chromatin remodeling complexes under mechanical strain

Direct evidence of ATP-dependent chromatin remodeling under mechanical load in periodontal or dental stem cells is sparse; where available, oral data are presented first, followed by conceptual insights from other mesenchymal systems. Mechanical forces regulate chromatin organization not only by physically deforming the nucleus but also by modulating ATP-dependent chromatin remodeling complexes that control DNA accessibility. Complexes such as SWI/SNF and ISWI reposition or evict nucleosomes to expose or occlude regulatory elements, enabling rapid transcriptional adaptation under strain. SWI/SNF (also known as the BAF complex) acts as a mechanosensitive switch: under low tension, ARID1A–SWI/SNF restrains YAP/TAZ activity; under high mechanical strain or on rigid substrates, this interaction is disrupted, releasing YAP/TAZ to activate target genes (Chang et al., 2018). The ISWI family similarly responds to mechanical cues, sliding nucleosomes along DNA to increase local chromatin fluidity and permit transcription factor access (Battilana et al., 2021). Consistent with this, mechanical stress increases overall chromatin accessibility: mesenchymal stem cells on stiff substrates exhibit elevated histone acetylation and reduced chromatin compaction, in part due to upregulation of HATs and downregulation of HDACs (Engler et al., 2006; Killaars et al., 2020; Heo et al., 2015; Maki et al., 2021; Heo et al., 2016; Killaars et al., 2019; Damodaran et al., 2018). Compressive forces likewise modulate the levels of chromatin-modifying enzymes, suggesting biochemical tuning of remodeler activity under load.

Mechanotransduction pathways such as Rho–ROCK signaling and Ca^2+^ influx converge on chromatin regulators. These inputs can drive nuclear import of histone modifiers, including mechanosensitive HDAC3 via deformed nuclear pores (Le et al., 2016; Nava et al., 2020), and can post-translationally modify remodeler subunits to alter their activity. During mechanically induced osteogenic differentiation, SWI/SNF is recruited to promoters of early response genes, where it facilitates nucleosome eviction and rapid gene activation (Chang et al., 2018; Battilana et al., 2021). In oral stem-cell populations, although direct evidence is still limited in these documents, it is reasonable to infer that ATP-dependent remodelers are similarly mobilized under tensile strain to open chromatin at osteogenic or odontogenic loci.

Mechanical forces also act at higher levels of genome architecture. Via the LINC complex and the nuclear lamina, external load alters the distribution of heterochromatin and euchromatin and reshapes 3D genome topology (Walker et al., 2021; Walker et al., 2022). Disrupting these nuclear mechanical connections prevents force-induced epigenetic changes (Walker et al., 2021; Walker et al., 2022), while specific chromatin-modifying enzymes help encode a “mechanical memory” of prior strain (Heo et al., 2015; Turner, 2002; Hathaway et al., 2012; Fan et al., 2017; Peng et al., 2017; Scott et al., 2023; Scott et al., 2024). Despite these advances, important questions remain - including how distinct force modes (compression vs. shear) differentially affect chromatin, and how universal these mechanisms are in oral stem cells (Hil et al., 2008; Perhonen et al., 1985; Tingare et al., 2013). Continued development of high-resolution, force-coupled Hi-C and live-cell nucleosome imaging is expected to define how mechanical loading rewires enhancer–promoter topology and chromatin accessibility in real time (Lammerding et al., 2004; Lyon et al., 2015). Ultimately, integrating biomechanics with epigenetic control offers a framework for precision regenerative dentistry, in which tuning the mechanical environment could epigenetically direct oral stem cells toward desired lineages (Yamazaki et al., 1998; Reed et al., 2014; White et al., 2006; Ruwhof and van der Laarse, 2000; Saucerman et al., 2019).

## 5 Non-coding RNAs and RNA modifications in mechanical signaling

In PDLSCs, a force-responsive long noncoding RNA (lncRNA) network has been described. Cyclic stretch downregulates SNHG8, which interacts with the Polycomb complex component EZH2; reduced EZH2 activity and the associated decrease in H3K27me3 correlate with enhanced osteogenic gene expression and mineralization (Zhang et al., 2022). Conversely, SNHG1 recruits EZH2 to the KLF2 promoter, increasing H3K27me3 and silencing this osteogenesis-promoting factor, thereby suppressing differentiation (Li Z. et al., 2020). Together, these findings identify EZH2-centered chromatin repression as a mechano-responsive switch governing PDLSC osteogenesis (Zhang et al., 2022; Li Z. et al., 2020) (Supplementary Table S3).

### 5.1 Mechanical forces orchestrate the epigenetic landscape of oral stem cell fate

Mechanical cues reshape the epigenetic landscape of oral stem cells, directing lineage commitment and establishing long-term “mechanical memory” through coordinated transcriptional and chromatin remodeling events (Alisafaei et al., 2019; Ferrari and Pesce, 2020). Among post-transcriptional regulators, mechanosensitive microRNAs (miRNAs) act as key intermediaries that couple physical forces to epigenetic control. In human PDL cells (PDLCs), cyclic tensile strain or orthodontic loading markedly downregulates miR-195-5p; this reduction promotes osteogenic differentiation by relieving repression of its targets WNT3A, FGF2, and BMPR1A (Chang et al., 2017).

Another pivotal miRNA, miR-21, behaves as a “mechanical memory keeper.” Stiff substrates sustain miR-21 expression through MRTF-A–dependent activation, and silencing miR-21 erases memory of prior mechanical conditioning (Ferrari and Pesce, 2020). miR-21 also regulates epigenetic enzymes: its inhibition increases DNMT1, DNMT3A, and TET2, suggesting that miR-21 normally suppresses DNA methylation machinery to modulate methylation dynamics (Sabry et al., 2023). In addition, miR-146a is force responsive and interfaces with chromatin modifiers during inflammatory signaling, implying a role in shaping histone modification patterns under load (Liao et al., 2023).

Together, these findings indicate that mechanosensitive miRNAs integrate mechanical inputs with epigenetic regulation. By tuning DNA methylation and histone modification programs, they stabilize osteogenic and inflammatory gene expression states in oral stem cells and help convert transient forces into heritable fate decisions (Ferrari and Pesce, 2020; Chang et al., 2017; Sabry et al., 2023; Liao et al., 2023).

### 5.2 LncRNAs as scaffolds for epigenetic complexes under force

Mechanical stimulation alters lncRNA expression in mesenchymal stem cells, enabling these transcripts to guide chromatin-modifying enzymes to specific genomic loci in a force-dependent manner (Ferrari and Pesce, 2020). HOTAIR exemplifies this mechanism: it functions as a modular scaffold that binds PRC2 at its 5′end and the LSD1/CoREST demethylase complex at its 3′end, thereby coordinating H3K27me3 deposition and removal of activating histone marks to enforce transcriptional silencing of developmental genes and promote osteogenic differentiation (Price et al., 2021).

MALAT1, a nuclear-retained lncRNA, similarly interacts with PRC2 components (EZH2, SUZ12, EED) to promote H3K27me3-mediated repression (Amodio et al., 2018). MALAT1 is mechanosensitive, with its levels influenced by shear stress and matrix stiffness, suggesting that mechanical inputs may elevate MALAT1 to silence inhibitory or inflammatory genes and thereby favor pro-osteogenic and pro-reparative programs (Caron et al., 2025).

Other lncRNAs, including Meg3 and H19, also respond to mechanical stress and act as guides that recruit histone methyltransferases or deacetylases to target promoters, coupling force exposure to site-specific epigenetic remodeling (Ferrari and Pesce, 2020; Piccoli et al., 2017). Collectively, these lncRNAs operate as force-regulated scaffolds that translate biomechanical cues into locus-specific chromatin states and lineage specification.

### 5.3 Mechanical control of the “epitranscriptome” (m6A and m5C RNA modifications)

Mechanical signals extend beyond DNA and histones to remodel the epitranscriptome. RNA modifications such as N^6^-methyladenosine (m^6^A) and 5-methylcytosine (m^5^C) influence transcript stability and translational output, thereby controlling the availability of key regulators. Recent work shows that mechanotransduction can reshape m^6^A patterns through metabolism-driven pathways. Li et al. demonstrated that an adaptable extracellular matrix (ECM) hydrogel mimicking intramembranous ossification elevates succinate levels in mesenchymal stem cells; succinate inhibits the m^6^A demethylase FTO, thereby favoring METTL3-mediated methylation of Runx2 mRNA, enhancing its translation, and promoting osteogenic differentiation under mechanically relevant conditions (Li et al., 2025).

Mechanical stress also modulates m^6^A writers and readers that act on transcripts encoding chromatin regulators. In cardiac hypertrophy, loss of the m^6^A-related factor METTL5 disrupts m^6^A-dependent translation control, causing aberrant accumulation of SUZ12, a PRC2 subunit, and altering chromatin state under mechanical load (Han et al., 2022). Moreover, m^6^A “reader” proteins such as YTHDF1 and YTHDF3 enhance translation of methylated transcripts, potentially including those that encode DNA- and histone-modifying enzymes, thereby linking RNA methylation to chromatin reprogramming in mechanically challenged cells (Geula et al., 2015; Wang et al., 2016; Zheng et al., 2013; Kumari et al., 2022; Wang X. et al., 2015).

Beyond m^6^A, m^5^C provides an additional layer of force-responsive epigenetic coupling. The m^5^C methyltransferase NSUN2 installs m^5^C marks and, via the m^5^C-binding protein ALYREF, engages Jarid2/Ezh2 to recruit PRC2 to chromatin, forming an NSUN2–PRC2 axis that can position PRC2 at defined genomic loci in an m^5^C-dependent manner (Hu et al., 2025). Although this NSUN2–PRC2 pathway has not yet been directly mapped under mechanical loading, it is plausible that mechanically regulated changes in NSUN2 abundance or localization could redirect PRC2 targeting, thereby reshaping histone methylation and transcriptional programs.

In sum, mechanical forces can dynamically rewire RNA methylation networks - including m^6^A- and m^5^C-dependent control of transcript stability and translation - to regulate both epigenetic enzymes and lineage-specifying transcription factors. Through this epitranscriptomic layer, biomechanical input is converted into chromatin remodeling and fate specification in oral stem cells (Li et al., 2025; Han et al., 2022; Geula et al., 2015; Wang et al., 2016; Zheng et al., 2013; Kumari et al., 2022; Wang X. et al., 2015; Hu et al., 2025).

## 6 Mitochondrial metabolism: bridging mechanics and epigenetics

### 6.1 Mechanoregulation of mitochondrial function

Mechanical forces dynamically regulate cellular metabolism by linking extracellular matrix (ECM) mechanics to mitochondrial activity. Cells sense ECM stiffness through integrin-based and YAP/TAZ-dependent mechanotransduction pathways, which reprogram metabolic flux and energy production (Park et al., 2020). When cells are transferred from a stiff to a soft substrate, cytoskeletal relaxation suppresses glycolysis, coupling ATP output to the mechanical properties of the environment (Park et al., 2020). Conversely, stiffer matrices activate MAPK–YAP signaling, enhancing aerobic glycolysis and ATP synthesis to satisfy elevated energetic demands (Verbakel and Boer, 2025).

In mesenchymal stem cells (MSCs), increased matrix rigidity promotes both glycolysis and oxidative phosphorylation (OXPHOS), together with enhanced antioxidant defense, indicating a global upregulation of mitochondrial metabolism during osteogenic differentiation (Na et al., 2024). These observations show that force-induced cytoskeletal remodeling modulates mitochondrial function by regulating key metabolic enzymes, mitochondrial biogenesis, and tricarboxylic acid (TCA) cycle flux (Park et al., 2020; Verbakel and Boer, 2025; Na et al., 2024).

Crucially, this metabolic rewiring provides a direct link from mechanics to epigenetics. Force-dependent shifts in mitochondrial activity alter pools of metabolites such as acetyl-CoA, αKG, S-adenosylmethionine (SAM), and NAD^+^, which act as cofactors for chromatin-modifying enzymes (Park et al., 2020). Thus, mechanoregulation of mitochondrial function establishes a metabolic–epigenetic axis through which physical forces can be converted into durable changes in gene expression (Park et al., 2020; Verbakel and Boer, 2025; Na et al., 2024).

### 6.2 Metabolites as epigenetic substrates and co-factors

Mitochondria-derived metabolites serve as a biochemical bridge between metabolism and the epigenome by acting as substrates or cofactors for chromatin-modifying enzymes. Acetyl-CoA donates acetyl groups for histone acetylation and thereby links glucose metabolism to gene activation. Through ATP-citrate lyase (ACL), citrate is converted to acetyl-CoA; growth factor–driven glycolysis increases ACL activity, elevating histone acetylation and promoting transcriptional activation. Conversely, nutrient deprivation or inhibition of acetyl-CoA synthesis reduces global histone acetylation and drives cells away from a stem-like state (Wellen et al., 2009).

αKG generated through the tricarboxylic acid cycle and glutamine metabolism, is an obligate cofactor for Jumonji histone demethylases and TET DNA demethylases. A high αKG/succinate ratio promotes DNA and histone demethylation, maintains open chromatin, and supports pluripotency, whereas succinate accumulation inhibits these demethylases and biases cells toward differentiation (Carey et al., 2015).

S-adenosylmethionine (SAM), produced by one-carbon metabolism that is tightly coupled to mitochondrial amino acid and folate pathways, supplies methyl groups for DNA and histone methyltransferases. In embryonic stem cells, threonine catabolism sustains SAM levels; depletion of threonine lowers SAM, reduces H3K4me3, and compromises self-renewal capacity (Shyh-Chang et al., 2013). Enzymes such as NNMT can further drain SAM, thereby tuning the cell’s methylation potential (Shyh-Chang et al., 2013).

Finally, NAD^+^ links mitochondrial redox state to chromatin structure by serving as a required co-substrate for sirtuin deacetylases. High NAD^+^ enhances Sirt1-dependent histone deacetylation and chromatin tightening, whereas an elevated NADH/NAD^+^ ratio diminishes sirtuin activity and favors hyperacetylated chromatin (Wu et al., 2022; Yang et al., 2022).

Together, acetyl-CoA, αKG, SAM, and NAD^+^ convert metabolic and mechanical inputs into epigenetic outputs. Because their levels are shaped by mitochondrial function, these metabolites directly regulate the efficiency of chromatin-modifying enzymes and allow metabolic state to be encoded in the epigenetic landscape (Wellen et al., 2009; Carey et al., 2015; Shyh-Chang et al., 2013; Wu et al., 2022; Yang et al., 2022).

### 6.3 Intercellular mitochondrial transfer and cross-talk

Beyond producing metabolites, mitochondria themselves can move between cells, providing a mechanism to restore bioenergetic balance and potentially propagate metabolic states. Under stress, recipient cells can acquire functional mitochondria from donor cells via tunneling nanotubes (TNTs) or extracellular vesicles. In oral–neural contexts, DPSCs transfer mitochondria to stressed Schwann cells through TNT-like structures, markedly reducing oxidative stress and pyroptotic cell death in the recipient cells while promoting nerve regeneration (Zheng et al., 2025). Blocking gap junctions or mitochondrial function diminishes these effects, confirming that donated mitochondria drive the rescue response (Zheng et al., 2025). Notably, TNFα released by injured Schwann cells enhances mitochondrial donation from DPSCs, revealing a bidirectional stress-responsive feedback loop (Zheng et al., 2025). Through this exchange, DPSCs effectively deliver a metabolically “younger,” stress-resistant state that may indirectly influence the epigenetic landscape of recipient cells by altering pools of NAD^+^ or acetyl-CoA and thereby modulating sirtuin activity or histone acetylation. This in vivo-like evidence demonstrates that oral mesenchymal stem cells can transfer mitochondria to neighbouring stressed cells, thereby modulating metabolic and potentially epigenetic states in the recipient. (Islam et al., 2012). In non-oral models, bone marrow mesenchymal stem cells donate functional mitochondria to damaged pulmonary alveolar cells, thereby restoring respiratory function and limiting acute injury. These BM-MSC–to-alveolar transfer studies provide broader context for stress-induced mitochondrial donation but do not constitute direct evidence in oral tissues (Islam et al., 2012).

Overall, intercellular mitochondrial transfer represents a shift in how cell–cell communication is understood: cells can exchange organelles, not just soluble signals, to buffer metabolic stress and coordinate regeneration (Zheng et al., 2025; Islam et al., 2012).

### 6.4 Mechanotransduction-driven metabolic-epigenetic axis

Mechanical signals not only reprogram mitochondrial activity but also control the availability of key metabolites that feed chromatin-modifying enzymes. On stiff matrices, cytoskeletal tension and YAP/TAZ activation drive glycolytic and glutaminolytic gene programs; inhibiting YAP reduces mitochondrial ATP production and oxidative phosphorylation (Fabiano et al., 2025; Wu et al., 2025; Liu et al., 2020; Enzo et al., 2015). Cytoskeletal tension also activates AMPK, which recruits GLUT1 to the membrane and liberates glycolytic enzymes such as phosphofructokinase-1 and aldolase from the actin cytoskeleton. While AMPK-dependent phosphorylation of PFKFB3 and endothelial nitric-oxide synthase further amplifies glycolytic flux (De Bock et al., 2013; Zhang et al., 2006; Doménech et al., 2015). These mechanotransduction pathways increase pools of acetyl-CoA and α-ketoglutarate via upregulation of ATP-citrate lyase and glutaminase, respectively, while also influencing one-carbon metabolism and NAD^+^/NADH balance. Because acetyl-CoA donates acetyl groups for HATs, α-ketoglutarate is a cofactor for Jumonji histone and TET DNA demethylases, S-adenosylmethionine (SAM) supplies methyl groups for methyltransferases, and NAD^+^ is required by sirtuin deacetylases, force-dependent changes in these metabolites directly bias histone acetylation, methylation and demethylation (Wu et al., 2025; Ouyang et al., 2025).

Most mechanometabolic studies have been performed in fibroblasts, epithelial or cancer cells; direct evidence that mechanical cues regulate metabolite pools and epigenetic writers/erasers in oral mesenchymal stem cells is sparse (Luo et al., 2022). Future work should employ stable-isotope tracing, metabolomics and single-cell multi-omics to define how YAP/TAZ, MAPK and cytoskeletal tension influence acetyl-CoA, α-ketoglutarate, SAM and NAD^+^ in mechanically loaded dental stem cells. Integrating biomechanical design (e.g., scaffold stiffness, dynamic loading) with metabolic modulators may yield precision strategies to steer pulp and periodontal regeneration.

## 7 Synthesis, challenges, and future perspectives

### 7.1 Single-cell epigenomic approaches in oral stem cells

Single-cell epigenomic technologies are transforming how we understand gene regulation in dental and oral stem cells. Epigenetic regulation encompasses chromatin remodeling, DNA methylation, histone modifications and RNA modifications (Zhang et al., 2020; Sun et al., 2023; Trixl and Lusser, 2019; Gopinathan et al., 2013), and these processes influence the differentiation of DPSCs, stem cells from the SCAPs, SHEDs, PDLSCs and other oral mesenchymal stem-cell populations (Shi et al., 2020). Traditional studies often relied on bulk assays, but single-cell methods now enable the mapping of epigenetic landscapes at cellular resolution. Single-cell assay for transposase-accessible chromatin (scATAC-seq) and multiome approaches that simultaneously capture RNA and chromatin accessibility are now being used in craniofacial tissues. In a recent multiome study of mouse secondary palate development, researchers profiled chromatin accessibility and gene expression simultaneously in >36,000 cells. They reconstructed trajectories in cranial neural-crest–derived multipotent cells, linking open chromatin to gene-expression changes and identifying lineage-determining transcription factors such as SHOX2 and MEOX2 (Yan F. et al., 2024). This work provides an example of how scATAC-seq plus scRNA-seq can chart epigenetic and transcriptional dynamics during dental mesenchymal differentiation.

Cleavage-under-targets-and-tagmentation (CUT&Tag) is a newer antibody-guided chromatin profiling method that can work with small cell numbers or single cells. A recent review of post-translational modifications in the oral micro-environment noted that spatial CUT&Tag mapping was adapted to regenerating periodontal tissue, revealing interwoven domains of H3K9ac and H3K27me3 that corresponded to zones of active PDLSCs; by contrast, early oral squamous cell carcinoma lesions displayed discrete clusters of H3K27me3-rich cells (Bartosovic and Castelo-Branco, 2023). Such spatial–epigenomic methods preserve tissue architecture while resolving histone-modification patterns.

Single-cell epigenomics has also been integrated with lineage-tracing studies of dental stem cells. In a 2025 Science Advances paper, Ce Shan and colleagues combined scRNA-seq with CUT&Tag and spatial mapping to examine Cd24a+/Pax9+ dental stem cells during postnatal tooth development. They found that scRNA-seq and CUT&Tag together could delineate distinct features of these stem cells and their organization. CUT&Tag profiling of H3K4me3 across sorted Cd24a^+^/Pax9^+^ and Cd24a^−^/Pax9^−^ populations revealed cell-type-specific patterns-genes like Dvl2 showed broader H3K4me3 domains in Cd24a^+^/Pax9^+^ cells, whereas Pthlh and Irf5 exhibited subtype-specific patterns (Shan et al., 2025). These differences correlated with progenitor states versus differentiation into odontogenic or vascular lineages.

Developmental studies on mouse incisors further highlight how single-cell transcriptomics can be paired with CUT&Tag. An IADR abstract and associated GEO dataset report that deletion of the transcription factor Six1 perturbs transitions from dental ectomesenchyme to dental papilla. Researchers generated a single-cell atlas of incisor germs spanning bud to bell stages and used H3K27ac and SIX1 CUT&Tag to show that Six1 directly binds promoters of Dlx1, Dlx2 and Dlx5; its absence led to widespread epigenetic and transcriptional remodeling. Such integrative approaches link transcription factor binding, enhancer activity and cell-state transitions during tooth development.

Together, these examples illustrate that single-cell epigenomic methods—scATAC-seq, multiome sequencing, and CUT&Tag—are beginning to illuminate the gene-regulatory logic of oral stem cells. By resolving chromatin accessibility and histone-modification patterns at cellular resolution, they help identify lineage-specific enhancers, transcription factors and signaling pathways that drive dental stem-cell differentiation and regeneration, providing foundations for precision regenerative therapies.

### 7.2 Technical advances: single-cell multi-omics and live-cell imaging

Decoding this mechano-epigenetic network requires resolving cellular heterogeneity and tracking chromatin dynamics in real time. Single-cell multi-omics approaches, such as combined scRNA-seq and ATAC-seq, reveal transcriptional and chromatin-accessibility variation among mesenchymal subpopulations in mouse dental pulp, including differential expression of DNMTs, TET enzymes, and chromatin remodelers (Alisafaei et al., 2019). Future integration with assays such as scChIC-seq or CUT&Tag will allow simultaneous mapping of histone marks and gene expression in individual cells.

Complementarily, advanced live-cell imaging strategies-including FRET-based fluorescent biosensors-enable real-time visualization of histone acetylation, histone methylation, and chromatin compaction during controlled mechanical loading in DPSCs (Cheleschi et al., 2017; Wang et al., 2018; Elosegui-Artola et al., 2017; Jain et al., 2013; Roy et al., 2018). Together, these tools bridge static multi-omic snapshots with dynamic chromatin behavior, offering unprecedented insight into how biomechanical signals are encoded and maintained in the epigenome (Alisafaei et al., 2019; Cheleschi et al., 2017; Wang et al., 2018; Elosegui-Artola et al., 2017; Jain et al., 2013; Roy et al., 2018; Ke et al., 2018).

### 7.3 Therapeutic implications and precision dentistry

Insights into mechano-epigenetic regulation are driving new regenerative and orthodontic strategies. One emerging approach is to pair defined mechanical cues with targeted epigenetic modulators to restore or enhance stem cell function. In PDLSCs compromised by diabetic or inflammatory stress, the DNA methyltransferase inhibitor 5-aza-dC reverses force- and stress-associated hypermethylation, reactivates Wnt/β-catenin signaling, and rescues mineralization capacity (Duncan et al., 2016). Similarly, in rat DPSCs, the histone deacetylase (HDAC) inhibitor SAHA elevates MMP-13 expression, enhances mineral nodule formation, and promotes cell migration, suggesting utility during guided pulp regeneration and orthodontic tooth movement (Duncan et al., 2016).

Engineered biomaterials provide a complementary, localized route for mechano-epigenetic control. Smart hydrogels with tunable stiffness can dynamically modulate nuclear mechanics and chromatin acetylation, effectively mimicking physiologic changes in tissue rigidity (Song et al., 2020). Embedding controlled-release nanoparticles or surface-bound epigenetic drugs (such as 5-aza-dC or SAHA) into these scaffolds could concentrate chromatin reprogramming within defect sites, while developmentally inspired matrix architectures may help steer DPSC differentiation (Song et al., 2020).

Key challenges remain. Patient-to-patient variability, context-dependent responses to force, and off-target effects of epigenetic drugs complicate translation. Replicating the complex *in vivo* mechanical milieu and ensuring the durability and safety of induced chromatin states are ongoing hurdles. Nonetheless, the rescue of osteogenesis by 5-aza-dC and the enhancement of mineralization by SAHA illustrate the promise of mechano-epigenetic precision dentistry-an approach that converges mechanobiology, single-cell epigenomics, and responsive biomaterials to enable next-generation dental regeneration (Duncan et al., 2016; Song et al., 2020).

## 8 Conclusion

Mechanical signals regulate the fate of oral stem cells through multi-layered epigenetic mechanisms, forming an integrated mechano-epigenetic network. In DPSCs, matrix stiffness simultaneously alters DNA methylation and histone states: stiff substrates suppress DNMT1, reducing global DNA methylation, whereas soft matrices enhance histone acetylation (e.g., H3K9Ac) and decrease repressive marks such as H3K27me3 (Cozzolino et al., 2016; Qu et al., 2018; Pennarossa et al., 2018; Li et al., 2017). Mechanical signals also directly modulate chromatin-modifying enzymes: on compliant matrices, HAT1 expression is upregulated while HDAC1/2 are downregulated, leading to globally elevated histone acetylation and transcriptional activation (Cozzolino et al., 2016).

Mechanosensitive long non-coding RNAs (lncRNAs) constitute another regulatory layer. Under cyclic strain, SNHG8 is induced in PDLSCs, where it inhibits the PRC2 methyltransferase EZH2 and reduces H3K27me3, thereby promoting osteogenic differentiation. Conversely, SNHG1 recruits EZH2 to silence osteogenic programs (Qu et al., 2018; Pennarossa et al., 2018; Li et al., 2017).

Membrane force sensors such as Piezo1 convert mechanical forces into intracellular Ca^2+^ and kinase signals, activating osteogenic/odontogenic transcription factors (Dong et al., 2025). In PDLSCs, mechanical stretch downregulates lncRNA SNHG8, and its loss decreases EZH2 and H3K27me3 deposition at lineage gene loci, thereby derepressing osteogenic genes (Hu and Fan, 2025; Zhang et al., 2022; Li Z. et al., 2020). Meanwhile, mechanical force upregulates the H3K27 demethylase KDM6B (JMJD3), removes repressive H3K27me3 marks, and activates Wnt signaling to promote mineralized differentiation (Ying et al., 2024). Classical epigenetic regulators (HATs/deacetylases, DNA methyltransferases, chromatin remodeling complexes) integrate with mechanotransduction pathways to co-regulate gene expression. Histone methylation (e.g., H3K4, H3K9, H3K27) is a critical node linking mechanical cues to gene regulation, with changes correlating with altered RUNX2 and Wnt activity (Hu and Fan, 2025; Zhang et al., 2022; McCreery et al., 2025; Huang et al., 2021). Furthermore, mechanical signals alter nuclear morphology and LaminA levels, modulating chromatin accessibility (McCreery et al., 2025). Non-coding RNAs (e.g., lncRNA FER1L4) act as mechano-effectors (Huang et al., 2021).

These findings open new avenues in regenerative dentistry and orthodontics. Scaffold/biomaterial design can be optimized to deliver mechanical signals that pre-program stem cell epigenomes, guiding cells toward desired lineages through nuclear tension and chromatin modulation (Lee et al., 2025). Combining mechanical therapy with epigenetic drugs (e.g., targeting EZH2 or histone deacetylases) may improve therapeutic outcomes (Hu and Fan, 2025). Future directions include *in vivo* validation, utilizing single-cell and spatial omics technologies to decipher heterogeneity, developing adaptive scaffolds with precise strain control, and exploring novel strategies such as “force-guided chromatin editing.”
