# Supplementary material for: Mechanical forces orchestrate the epigenetic landscape of oral mesenchymal stem/progenitor cell fate in dental and periodontal tissues
Source: Front Cell Dev Biol. 2026 Feb 27;14:1743397. doi: 10.3389/fcell.2026.1743397 (PMC12982463; doi:10.3389/fcell.2026.1743397)
Supplement: Supplementary file 1 [file DataSheet1.pdf]

Supplementary Table S1. DNA methylation-related changes under mechanical contexts (oral evidence)

System	Mechanical context (baseline explicitly defined)	Key methylation event	Functional outcome	Key references
hPDLSCs	Mechanical force vs static control	DNMT1/DNMT3B-mediated MIR31HG promoter hypermethylation	↓ proliferation; ↑ IL-6 under load	[117]
hPDLSCs	Stiff vs soft GelMA (as defined in study)	↑ DNMT activity / DNMT3B; ↑ global 5-mC (reported)	Osteogenic bias (RUNX2/ALP etc.)	[121]
hPDLSCs	High glucose vs normal glucose (pathologic context)	↑ DNMT / global hypermethylation	Impaired osteogenesis rescued by demethylation	[125]

Supplementary Table S2. Histone marks under mechanical cues (oral evidence prioritized)

Axis	Mechanical context	Cell system	Key finding	Key references
Global H3 acetylation / H3K9me3	Cyclic stretch vs static	PDL cells	↑ H3 acetylation; ↓ H3K9me3 (chromatin opening-like)	[128]
EZH2–H3K27me3	Compression vs no compression	hPDLSCs	EZH2 reduction is required mechanoresponse (mechanistic)	[130]
H3 acetylation–IL10	Force-stressed PDL fibroblasts ± HDAC/HAT perturbation	PDL fibroblasts	Acetylation-dependent IL10 induction	[129]

Supplementary Table S3. ncRNA under mechanical contexts (oral evidence only)

ncRNA	Mechanical context	Cell type	Epigenetic linkage	Outcome	Key references
SNHG8	Cyclic stretch vs static	hPDLSCs	EZH2/H3K27me3-related axis	Promotes osteogenesis (as reported)	[82]
SNHG1	Force-associated regulation (study-defined)	PDLSCs	EZH2 recruitment to KLF2 promoter	Inhibits osteogenesis	[83]
miR-195-5p	Mechanical loading (study-defined)	PDLCs	Targets WNT3A/FGF2/BMPR1A (pathway; not "epigenetic axis")	Promotes osteogenesis	[177]
